# The Diagnosis of Gall Stones

**Published:** 1902-01

**Authors:** 


### The Diagnosis of Gall Stones.

Under the above title Dr. Charles G. Stockton has an interesting article in a recent number of *American Medicine* in which he reports a series of cases to illustrate the close relationship existing between gall stones and cholecystitis and to demonstrate the symptomatology of several varieties of gall stone disease. He concludes the article as follows: "There are a few general remarks which should be enumerated before closing: First, cholecystitis is often mistaken for gastralgia. I confess to having made the error, and in rare instances the differentiation is difficult. Second, we must remember the curious signs of transient pyloric obstruction that follow adhesions of the liver and gall bladder and the lower end of the stomach. Third, we must remember that jaundice is a far from necessary accompaniment of cholelithiasis. It is true that it often offers important evidence, especially as to the location of the stone, but when present, it is often the result of angiocholitis, and, when intermittent, it probably depends upon the presence of a stone in the terminus of the cystic duct, or a stone free in the choledochus. When a steadily increasing and persistent jaundice is met, it is safe to attribute the cause to outside pressure exercised upon the common duct by growth in the head of the pancreas or elsewhere, and not by stone in the biliary ducts. If asked to name the chief symptoms of gall stone disease—which include, of course, those of the attending cholecystitis—we may say they are: First, paroxysmal pain; second, tenderness below the junction of the ninth rib and cartilege; third, vomiting; fourth, ague-like fever; fifth, jaundice; sixth, the formation of tumor; seventh, collapse; eighth, the passage of calculi in the stools." R.

